# Geographical Detector-Based Risk Assessment of the Under-Five Mortality in the 2008 Wenchuan Earthquake, China

**DOI:** 10.1371/journal.pone.0021427

**Published:** 2011-06-27

**Authors:** Yi Hu, Jinfeng Wang, Xiaohong Li, Dan Ren, Jun Zhu

**Affiliations:** 1 School of Earth and Mineral Resource, China University of Geosciences, Beijing, China; 2 State Key Laboratory of Resources and Environmental Information System, Institute of Geographic Sciences and Natural Resources Research, Chinese Academy of Sciences, Beijing, China; 3 National Office for Maternal and Child Health Surveillance, West China Second Hospital, Sichuan University, Chengdu, Sichuan, China; Kenya Medical Research Institute - Wellcome Trust Research Programme, Kenya

## Abstract

On 12 May, 2008, a devastating earthquake registering 8.0 on the Richter scale occurred in Sichuan Province, China, taking tens of thousands of lives and destroying the homes of millions of people. Many of the deceased were children, particular children less than five years old who were more vulnerable to such a huge disaster than the adult. In order to obtain information specifically relevant to further researches and future preventive measures, potential risk factors associated with earthquake-related child mortality need to be identified. We used four geographical detectors (risk detector, factor detector, ecological detector, and interaction detector) based on spatial variation analysis of some potential factors to assess their effects on the under-five mortality. It was found that three factors are responsible for child mortality: earthquake intensity, collapsed house, and slope. The study, despite some limitations, has important implications for both researchers and policy makers.

## Introduction

On May 12th, 2008, at 2:28 p.m. local time, an earthquake registering 8.0 on the Richter scale hit the north-western part of Sichuan Province, China, with the epicenter in Wenchuan County. The devastating earthquake claimed more than 69,000 lives, many of which were children, particularly children less than five years old [1]. Child health development, as an essential part of global economic and social progress, has received wide attention from the international community. Particularly, the under-five mortality is one important indicator of a country or district's health level.

As part of ongoing efforts to understand spatial patterns and epidemiological characteristics of child mortality and to help take preventive measures in reconstruction, potential risk factors associated with earthquake-related child mortality need to be identified. Previous epidemiologic studies on earthquake-related mortality had identified associations between seismic deaths and structural damage in earthquakes [2], [3], [4], [5]. The effect of physical factors on mortality had also been reported [6], [7], [8]. The demographic patterns of mortality due to the immediate or prolonged effects of earthquakes, however, were inconsistent in previous studies.

Numerous risk assessment methods for deaths and injuries have been proposed. Usually, an epidemiological method is used to measure risk factors. For example, a multivariable logistic regression model was used to analyze risk factors for deaths and injuries in an earthquake in Afyon, Turkey [6]. Armenian *et al*. used a univariate and multivariate analysis model for the same purpose for an earthquake in Armenia [9]. However, the previous models or methods involve many assumptions, e.g. homoscedasticity and normality, and violation of such assumptions can have a major impact on model validity. Moreover, it is difficult to interpret interactions in classic models and the inclusion of interactions when a study was not specifically designed to assess them can make it difficult to estimate the other effects in the model. Therefore, there is a need to develop suitable, if not better, techniques for assessing the risk of child mortality in the present study.

In this study, we use a method of geographical detector proposed by Wang *et al.* [10] of assessing association between the under-five mortality and risk factors by means of spatial variance analysis (SVA). The basic idea of SVA is to measure the degree to which the spatial distribution of health problem (e.g. mortality rate) is consistent with that of risk factors (e.g. earthquake intensity, population density, structural damage, etc). Four detectors (factor detector, interaction detector, risk detector, and ecological detector) using concept of Power of Determinant (*PD*) were proposed to assess the main and interactive effect of potential risk factors on health problem. They offer a novel approach to extracting the implicit interrelationships between risk factors and child mortality without any assumptions or restrictions with respect to explanatory and response variables, and they also recognize the spatial patterns of risk factors and child mortality which are difficult to model using classic epidemiological methods. The Geographical detectors are usable for both quantitative data and nominal data. The later can cause trouble with classic regression when there are too many categories [11]. We first identify and map the spatial distribution of the under-five mortality at the township level, and then report on other relevant physical, social, and structural factors, such as earthquake intensity, population density, and collapsed house. Finally, we employ the four detectors to assess the association between child mortality and those factors and make discussion in terms of the results.

## Methods

### Mortality data

National Office for Maternal and Child Health Surveillance provided under-five mortality data collected at township level in the Wenchuan earthquake and population of children under five in the middle of 2008. The under-five mortality was defined as earthquake-related deaths among children below the age of five, either part of the local population or floating population, a phrase that describes unprecedented migrant flows, moving from where they were born, mostly the underdeveloped area to developed area, looking for works or leading a life. Two types of mortality were classified: direct and indirect death. Direct deaths were defined as being caused by structural failure, by being struck or trapped by an object dislodged during the shaking, or by a fall during the earthquake; indirect deaths included those caused by the aftermath of earthquake damage such as fires and secondary geological disasters or traffic control failure like car collisions.

Our study area includes 21 earthquake-hit counties confirmed by the Ministry of Civil Affairs, which are located in northeastern Sichuan Province. There are, up to May 2009, a total of 934 earthquake-related deaths of children under five in 115 townships, with 683 cases of direct death and 251 cases of indirect death.

**Figure 1:**
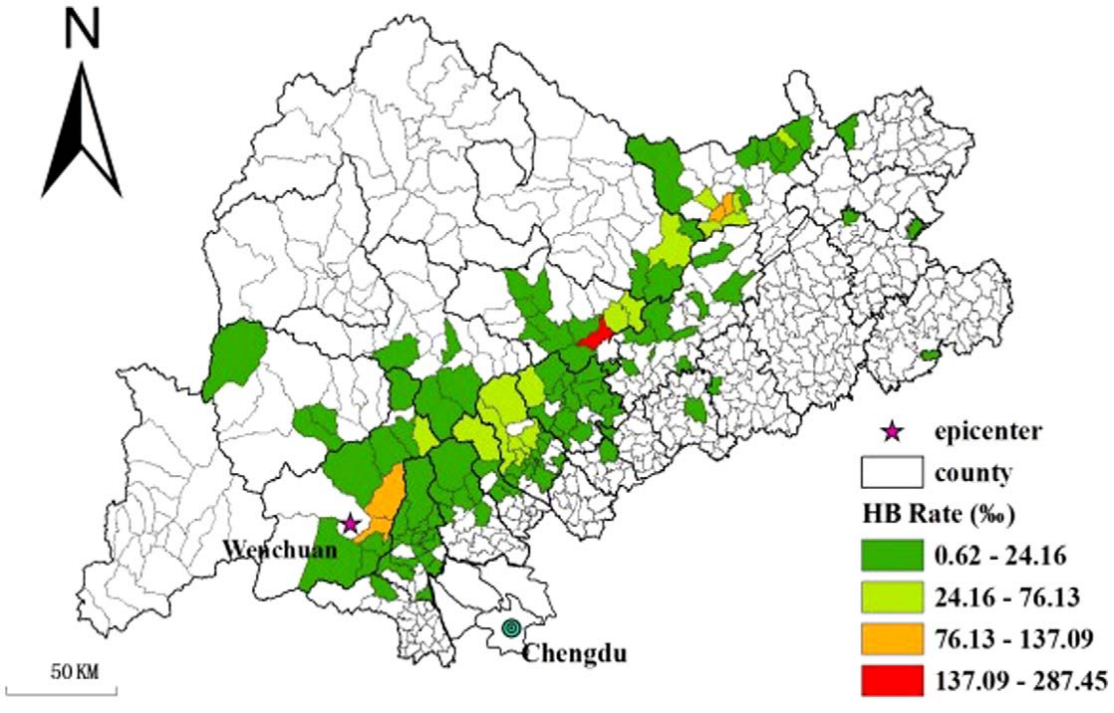
Hierarchical Bayesian smoothed under-five mortality rate at the township level in the Wenchuan earthquake (China, 2008). Thematic categories are based on the Jenks natural breaks method.

We calculated township-specific mortality rate for each of the 115 townships in the study area. A Hierarchical Bayesian (HB) model [12] was employed to address the problem of small population in explorative mapping of mortality rate in such small areas [13]. Details of how HB model was implemented are given in Text S1. Administrative code boundaries at the township scale in the form of shapefile were provided by the State Key Laboratory of Resources and Environmental Information Systems (LREIS) from the Institute of Geographic Sciences and Natural Resources Research (IGSNRR), Chinese Academy of Science (CAS). A final map of HB mortality rate using a Geographic Information System (GIS) environment is shown in Figure 1.

### Determinants of under-five mortality and their proxies

Key determinants leading to child mortality were diverse and complex, such as structural failure, object falling, fire, land slide, car collision, and etc. It is difficult to analyze those determinants directly as risk factors, e.g. it would be problematic to treat fire or car collision as risk factors. Actually, these complex determinants act through at least two geographic layers which are easy and convenient to implement with a GIS environment. These layers could be grouped as follows:

1. Physical factors that are spatially distributed. Magnitude measuring the energy released at the source of the earthquake and intensity measuring the strength of shaking produced by the earthquake at a certain location determine damages to building, residential structure, infrastructures and etc.; and the type of geomorphology like hill or mountain can determine whether secondary geological disasters would happen afterwards which could cause more destruction.
2. Social factors that are spatially distributed. The economic condition affects structural characteristics and construction materials of houses determining the resistance to earthquake damage and infrastructures that are associated with fire, traffic control failure, and etc.

**Figure 2:**
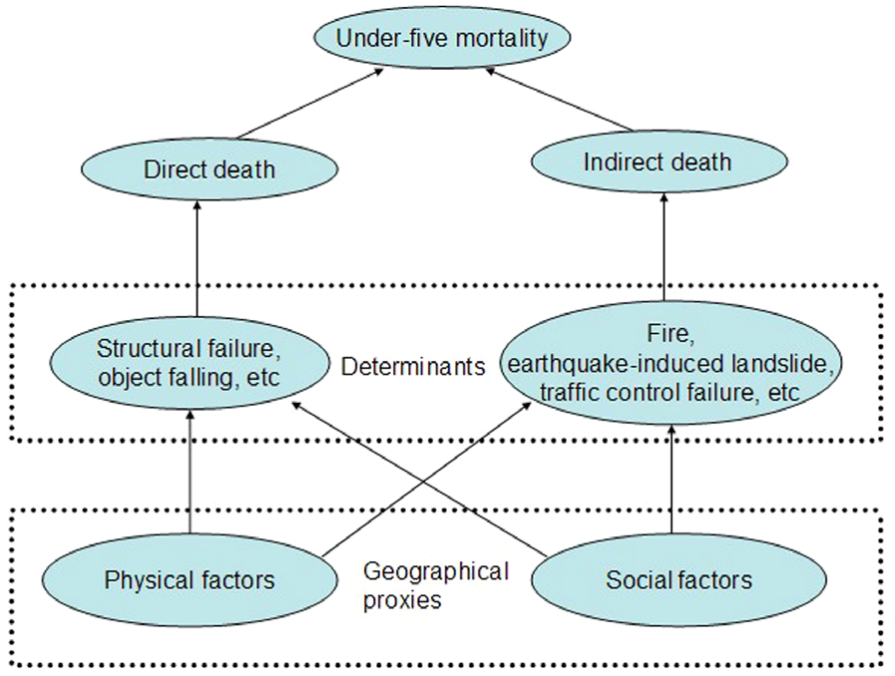
Relationship between determinants of mortality and their proxy variables.

**Figure 3:**
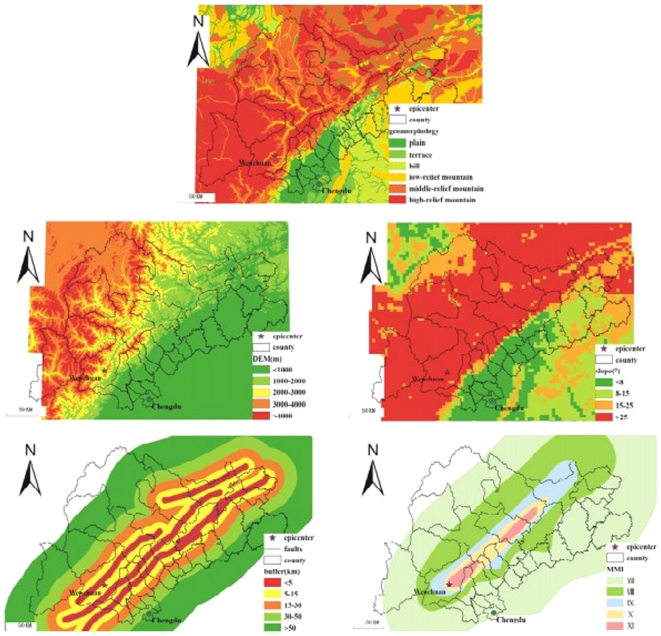
Maps of physical factors of the under-five mortality.

**Figure 4:**
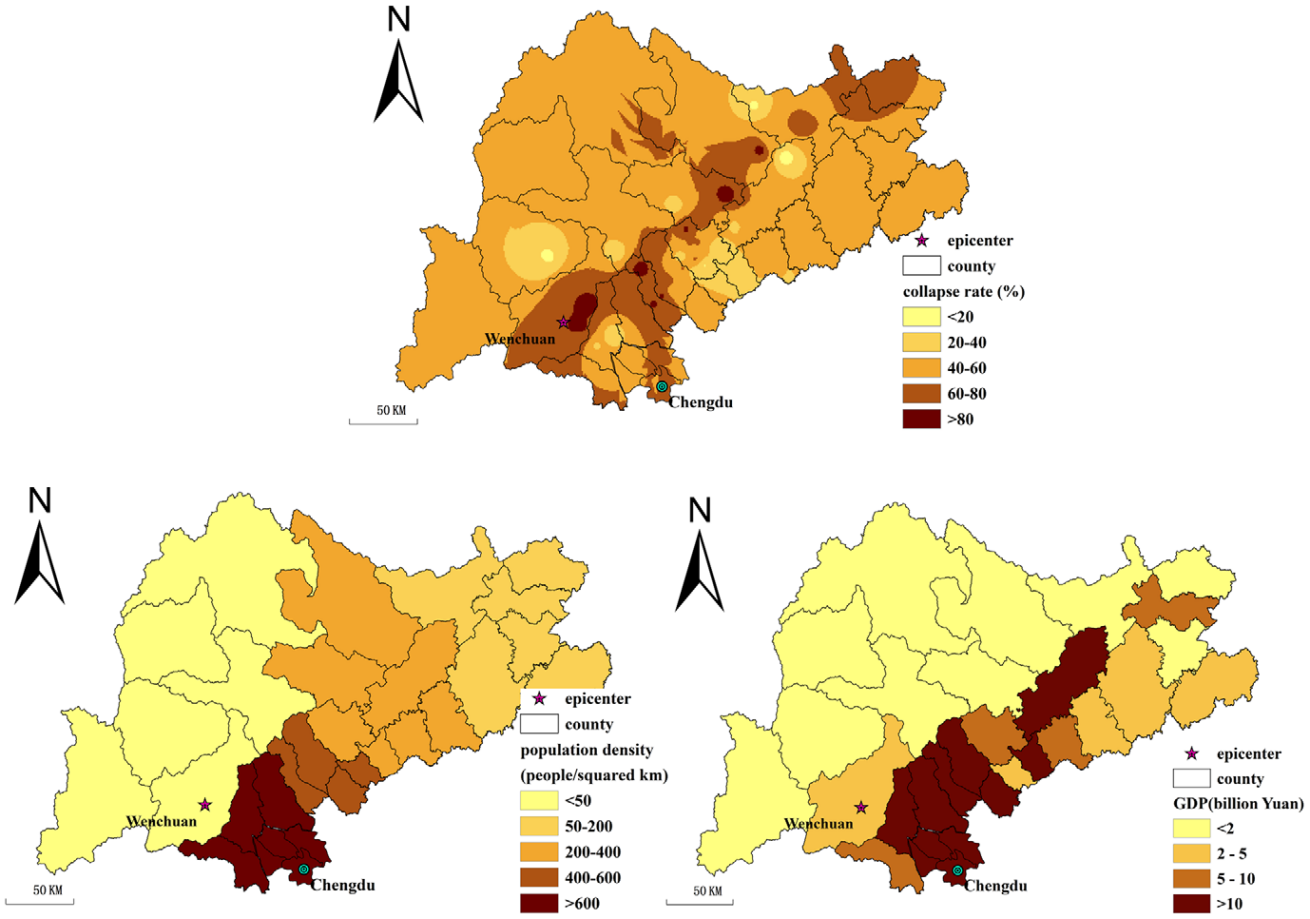
Maps of structural and social factors of the under-five mortality.

Figure 2 illustrates a conceptual framework that involves the direct determinants and their explicit geographical proxies. The physical and social factors were collected: elevation, slope, distance from township to fault, geomorphology, earthquake intensity, population density, and Gross Domestic Product (GDP). In addition, structural factor of collapsed houses at township level was collected. Figure 3 and Figure 4 present maps of these factors.

The topographic elevation was obtained from Digital Elevation Model (DEM). The DEM used in this study was derived from The Shuttle Radar Topography Mission (SRTM), an international project spearheaded by the U.S. National Geospatial-Intelligence Agency (NGA) and the U.S. National Aeronautics and Space Administration (NASA).

Slope is defined by a plane tangent to a topographic surface, as modeled by the DEM at a point [14]. Slope presents the percent change in elevation over a certain distance. The output slope can be calculated as either the percent of slope or degree of slope. In this study, degree of slope was chosen.

The geomorphology data were provided by the State Key LREIS of the IGSNRR of CAS. The geomorphology of the study area can be divided into seven classes: plain, terrace, hill, low-relief mountain, middle-relief mountain, high-relief mountain.

Earthquake intensity in the study area was taken from Modified Mercalli Intensity (MMI) scale map produced by China Earthquake Administration. The MMI scale is divided into twelve continuous categories [15]. The lower degrees of the MMI scale generally deal with the manner in which the earthquake was felt by people. The higher degrees are based on observed structural damage. The earthquake intensity in the study area ranged from VII to XI.

There are a series of thrust faults that affect the study area, for example, the Wenchuan-Maoxian, Yingxiu-Beichuan and Anxian-Guanxian faults. The earthquake intensity zones are elliptical, with those faults as major axes. Townships in the same intensity zone suffered the same devastating power described above, though some are closer to the epicenter. In addition, geologically, the rupturing of the crust around those faults is the main cause of structural damage. Consequently, we assumed that studying distance to faults to be more meaningful than studying distance to the epicenter. Using a GIS environment, some buffers around faults could be drawn.

Collapsed houses were measured by the proportion of completely collapsed houses among all registered houses. The completely collapsed house defined by the local government refers to house that had to be torn down and rebuilt for future occupancy. Collapsed house data at the township level were obtained from the Evaluation of Bearing Capacity of Resources and Environment (EBCRE) database, which was set up by IGSNRR, CAS, from the national reconstruction plan in 2009. Only 101 townships, however, in our study area have collapsed house data record, hence a method of inverse distance weighted interpolation [16] is used to predict the distribution of collapsed houses in the study area. The population density and GDP of 2007 were also sourced from EBCRE database as well.

### Statistical analysis

**Figure 5:**
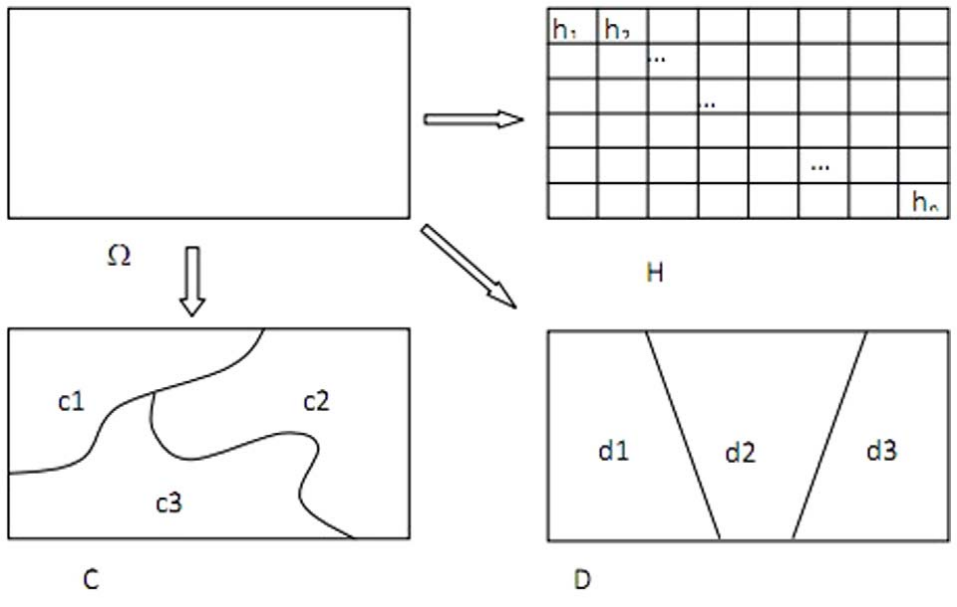
mortality distribution H and spatial patterns of suspect factors C and D in study area [**10**].

We assume that the child mortality would exhibit a spatial distribution similar to that of a risk factor if the risk factor leads to the mortality. The mechanism is quantified by power value as follows:

In our study area, Ω, (as shown in Figure 5) let the mortality be measured by the rates in grids, h_1_, h_2_, , h_n_; and let C and D be two potential risk factors associated with the mortality. Measurement of C and D can be continuous or categorical variable. Then, Ω is assumed to be stratified by the attribute of C and D, denoted as subareas {c1, c2, c3} and {d1, d2, d3}, respectively. For example, if the study area is stratified by geomorphology (measured by qualitative variable) then subareas denotes one type of geomorphology, or if the study area is stratified by elevation (measured by quantitative variable) then subareas can be obtained by any kind of discretization method depending on optimal classification or prior knowledge [17], [18].

The mortality layer H is overlaid by a potential factor layer, such as D for example. The average mortality rates, together with their variances, of the mortality in each subarea and in the study area Ω are denoted by 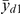,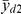,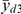,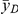, and Vard_1_, Vard_2_, Vard_3_, VarD, respectively. One hand, if the mortality is completely controlled by factor D, the rate will be identical everywhere in each of the subarea {d_1_, d_2_, d_3_}, and therefore, Vard_i_ (i = 1, 2, 3) will be zero. On the other hand, if the mortality is completely independent of factor D, the accumulated area's weighted dispersion variances of the rate of the subareas will be no different from the pooled area's weighted dispersion variances of the study area Ω. The mechanism is measured by the Power of Determinant (PD):



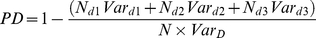
(1)where *N* and *N_di_* denote the areas of the study area Ω and the subarea di, respectively. If factor D completely controls the mortality *PD* equals 1; if factor D is completely unrelated to the mortality *PD* equals 0. Usually the value of *PD* lies in [0, 1]. The larger the value of *PD*, the greater is the impact of D on the mortality. Thus, the *PD* value quantifies the association between the under-five mortality and risk factors

Specially, the geographical detectors are composed of the following four detectors:

(1) Risk detector

The risk factor using t-value test compares differences in 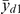,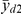, and 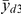 of factor D to check whether the average mortality rate in each subarea is statistically different when the study area is stratified by a potential risk factor D.

At this point, it is assumed that mortality occurs independently and identically over space [19]. According to the central limit theorem, the mean mortality occurrence is asymptotically normally distributed [20]. From the perspective of superpopulation, this mean is a single realization of an underlying process [21]; thus, the difference between two superpopulation means can be tested by student t. The superpopulation mean is estimated by the observed mean based on the ergodic assumption [22].

The geographical zones z (stratified by risk factor D) are ordered in descending order of risk 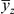. Either sample random variation or fundamental differences of superpopulations could result in difference between the means of two geographical zones which could be tested by the following statistic for two distributions having an unequal variance [23].



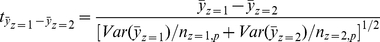
(2)where 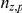 denotes the number of sample units 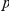 in zone 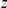
and 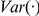
refers to variance. It is asymptotically distributed as Student's t with the degree of freedom (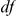) equal to



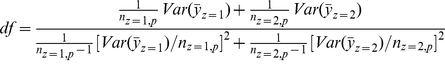
(3)


The null hypothesis is 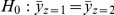. If 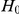 is rejected under a significant level 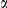
(usually 5%), it indicates that there is a significant difference between the mortality risk of zone 1 and 2.

(2) Factor detector

The factor detector quantifies the impact of a factor D on the observed spatial mortality pattern using the dispersion variance (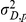) and the stratified population dispersion variance (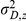). Therefore, PD value of factor D can be specifically expressed as follows:



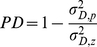
(4)




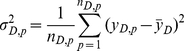
(5)




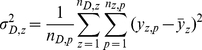
(6)where 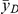 and 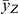
refer to average mortality rate within coverage of factor D and a specific zone stratified by D respectively.

(3) Interaction detector

The interaction detector quantifies interactive effect of two risk factors C and D, for example, by overlaying geographical layer C and D in GIS environment to form a new layer E, for example. The attribute of layer E is determined by combination of those of layer C and D. With the *PD* of layer C, D, and E, the interaction detector can deal with an interesting issue of whether two factors together have a stronger or weaker effect on mortality than they do independently.

(4) Ecological detector

The ecological detector based on F-value test compares C and D and explores whether C is more significant than D in controlling the spatial pattern of the mortality. If C is more likely than D to cause mortality over space, we would expect the dispersion variance of C (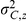) to be larger than that of D (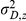). The test for this is:



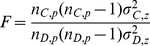
(7)where 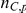 and 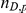
denotes the number of sample units
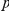
within coverage of C and D respectively.

This statistic is asymptotically distributed as 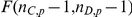 with 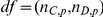.

Again, the null hypothesis is 
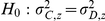
. If 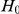 is rejected conditioned on a significant level 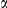
(usually 5%) and, it indicates that the effect of risk factor C on mortality is significantly different from that of D.

Another issue of implementing this method is to change the scale of mortality rate in the study area Ω to grid layer H. Usually, mortality rate is conveniently assigned to units selected by the human agent( postal, census, and administrative) [24], which is the township scale in our study. We use a method of even spatial discretization to downscale the mortality from the township scale to a grid layer H with unit square.

We developed a software to implement the four detectors, which can be freely downloaded at http://www.sssampling.org/GeoDetector.

## Results

The risk detector uncovers that the average mortality rates in each earthquake intensity zone (from VII to XI) are 3.223‰, 5.628‰, 19.565‰, 35.027‰, and 50.690‰ respectively and that they are statistically different (shown in table S1), indicating that higher mortality occurred in heavier intensity zone. Similar analysis of other risk factors can be conducted using the risk detector.

The factor detector discloses the influence of risk factors on the under-five mortality which is ranked by *PD* value as follows:

Earthquake intensity (0.446) > collapsed house (0.338) > slope (0.315) > population density (0.141) > DEM (0.116) > fault (0.063) > geomorphology (0.060) > GDP (0.048)

The ecological detector (shown in table S2) shows that differences of PD between earthquake intensity, collapsed house, and slope are not statistically significant, that differences between the rest factors are not statistically significant either, and that differences between any one of the first three factors and any one of the rest factors, however, are statistically significant. With the factor detector and the ecological detector, we find that earthquake intensity, collapsed house, and slope have strong effect on the under-five mortality, whereas the remaining factors have weak influence.

Joint impacts of two factors measured by PD value are shown in table S3 and can be compared with their separate impacts. The interactive effects between earthquake intensity and population density (0.584), earthquake intensity and slope (0.552), earthquake intensity and collapsed house (0.541), earthquake intensity and GDP (0.524), and earthquake intensity and DEM (0.475) are stronger than the effect of earthquake intensity (0.446) which has the strongest main effect on the under-five mortality, whereas the interactions between earthquake intensity and geomorphology (0.437) and earthquake intensity and fault (0.405) weaken the effect of earthquake intensity. Similarly, interactions between slope and other factors are fluctuating compared to main effect of slope. Collapsed house when interacting with any other factors, however, is even more devastating compared to itself (0.338) as a separate factor. Even of those factors with least impact, interactions between them enhance their separate effects on the mortality. These findings indicate that interaction between factors plays an important role in the under-five mortality.

## Discussion

In this study, we used four geographical detectors (GeoDetectors) to assess effects of some physical, social, and structural factors on the under-five mortality. We believe this method to be novel in that it extracts the interrelationships between health problem and risk factors by the correspondence of their spatial distribution and that it's easy to implement. This easy and efficient tool is extremely welcome in those undeveloped areas where health resources are limited in determinant detection for intervention and prevention.

Of more concern is which factor has the greatest role in the under-five mortality. With the four detectors we found that earthquake intensity, collapsed house, and slope were mainly responsible for the under-five mortality; that the higher MMI was the heavier mortality occurred, and so was collapsed house and slope; and that interactive effects between pairs of earthquake intensity, collapsed house, and slope are even stronger than their separate effects. Although elevation, fault, geomorphology, population density, and GDP were found to have weak effect on the under-five mortality they contributed a lot to the mortality when interacting with earthquake intensity, collapsed house, or slope, indicating the importance of these three factors.

Both earthquake intensity and collapsed house describe damage to residential houses and buildings. Trauma caused by partial or completes collapse of buildings and infrastructures is the overwhelming cause of death and injury in most earthquakes [25], [26]. The findings in our study highlight the importance of structural factor in causing lots of direct deaths. Masonry structure with timber roof and reinforced masonry structures were two types of structures which were widely used in most residential buildings, school buildings in the rural area, and some of factories, old residential and office buildings in the cities [27]. A lot of buildings like those completely collapsed or were heavily damaged in the earthquake. Some general reasons of their vulnerability are of long time use, lack of maintenance, poor redundancy of structural system, poor connections of pre-cast slabs, and etc.

One explanation for slope identified as an important contributor to child mortality could be secondary geological disasters triggered by earthquakes. Slope of topography is commonly regarded as directly related to landslide initiation; it is an important factor in landslide hazard analysis, and mudslides usually start on steep slopes and can be activated by natural disasters [28]. This indicates slope can be an agency to reflect the secondary geological disaster following the earthquake. The earthquake had a great impact on the local geological environment, resulting in large-scale disasters in Sichuan province like landslides, mud-rock flows, and quake lakes which leaded to lots of indirect death (as defined in this study). Preliminarily judging, about 1/3 of the whole Wenchuan earthquake losses were not by the direct result of the earthquake, but by the secondary geological disasters [29].

Our approach highlights a number of limitations and areas for further work. First, it is easy for the detectors to deal with qualitative factors, the values of which are determined by their nature or attributes, such as geomorphology. When dealing with quantitative factors, however, some prior knowledge is needed to discretize those quantitative variables. Arbitrary methods of discretization may not characterize actual association between risk factors and child mortality. Methodological problems related to the compilation of survey data sets constitute a further limitation to our analysis. Case data were collected in field surveys, with great attention given to minimizing missing data. For example, immediately after the earthquake, a three-level surveillance network, which was adapted from the national three-level maternal and children surveillance system, was established by the National Office of Maternal and Child Health Surveillance to collect under-five mortality data in the earthquake-hit areas. In this specialized system, interviewers at the county-level were recruited from local maternal and child health hospitals in the study areas; interviewers at the township-level were recruited from town hospitals, while interviewers at village-level were composed of village doctors, village chiefs, and village union directors. 80% of the interviewers in this system had been working on child health data collection for several years and had valuable experience in data collection. More importantly, the village-level interviewers had been living locally for many years and they were familiar with each family in their villages. However, all the interviewers were still arranged for to receive training on interview techniques. This system was similar to the national maternal and child health surveillance system that was used to survey under-five child mortality in China. A HB algorithm was employed to further reduce the uncertainty of the rate due to the small area. Collapsed house data in the EBCRE database were available for only 101 townships in the study area, leaving those of 14 townships unknown. To address this problem, we estimated collapsed house at these 14 townships using inverse weighted interpolation, which was proven to have a smaller Root Mean Square (RMS) error compared to other methods of interpolation using leave-one-out cross validation.

Despite some limitations, we still believe this study to be meaningful. Firstly, it is in an area of great public health interest and in which limited information was available. Most of previous earthquake-related studies mainly focused on general population. Seldom, however, did those pay attention to children under five. Psychologically and physically, children under five are largely dependent on their families, lack the ability to protect themselves in response to sudden disasters, and thus are more vulnerable to natural disasters like earthquakes. Secondly, results from this study help researchers to understand the epidemiological characteristics and spatial patterns of child mortality and provide clues to further studies. Finally, the implications from this study give to policy makers some clues to how and where to reconstruct new townships after earthquakes. Specifically, with regard to structural factor, the local government can alter building design practices in earthquake-prone areas and strictly supervise the resistant quality of buildings to ensure the structures' resistance to earthquakes. What's more, slope is a top priority when considering new sites for habitation. A topographic slope of 15° to 25° was the threshold for landslides and mud-rock flows in Sichuan Province [30]. It would be better to choose new township sites with a topographic slope below 15°. In addition, consideration of the slope of the land is important not only to minimize risks form natural hazards like earthquake and landslides, but to reduce construction costs and impacts of proposed development on natural resources such as soil, vegetation, and water systems.

## Supporting Information

Table S1 (DOC)

Table S2 (DOC)

Table S3 (DOC)

Text S1 Appendix.
(DOC)
