# Supplementary material for: Geographical Detector-Based Risk Assessment of the Under-Five Mortality in the 2008 Wenchuan Earthquake, China
Source: PLoS One. 2011 Jun 27;6(6):e21427. doi: 10.1371/journal.pone.0021427 (PMC3124508; doi:10.1371/journal.pone.0021427)
Supplement: Table S1 — (DOC) [file pone.0021427.s001.doc]

Table S1 Statistically significant difference of average under-five mortality rate between five earthquake intensity zones

| Difference | Ⅶ | Ⅷ | Ⅸ | Ⅹ | Ⅺ |
| --- | --- | --- | --- | --- | --- |
| Ⅶ |  |  |  |  |  |
| Ⅷ | Y |  |  |  |  |
| Ⅸ | Y | Y |  |  |  |
| Ⅹ | Y | Y | Y |  |  |
| Ⅺ | Y | Y | Y | Y |  |

Y means the difference of influence between the two factors is significant with the confidence of 95%, and N means not.
