# Supplementary material for: Geographical Detector-Based Risk Assessment of the Under-Five Mortality in the 2008 Wenchuan Earthquake, China
Source: PLoS One. 2011 Jun 27;6(6):e21427. doi: 10.1371/journal.pone.0021427 (PMC3124508; doi:10.1371/journal.pone.0021427)
Supplement: Table S2 — (DOC) [file pone.0021427.s002.doc]

Table S2 Statistically significant difference of influence of risk factors on the under-five mortality

| Difference | intensity | collapse | slope | density | DEM | fault | geomorphology | GDP |
| --- | --- | --- | --- | --- | --- | --- | --- | --- |
| intensity |  |  |  |  |  |  |  |  |
| collapse | N |  |  |  |  |  |  |  |
| slope | N | N |  |  |  |  |  |  |
| density | Y | Y | Y |  |  |  |  |  |
| DEM | Y | Y | Y | N |  |  |  |  |
| fault | Y | Y | Y | N | N |  |  |  |
| geomorphology | Y | Y | Y | N | N | N |  |  |
| GDP | Y | Y | Y | N | N | N | N |  |

Intensity: earthquake intensity; collapse: collapsed house; density: population density; Y means the difference of influence between the two factors is significant with the confidence of 95%, and N means not.
