# Supplementary material for: Geographical Detector-Based Risk Assessment of the Under-Five Mortality in the 2008 Wenchuan Earthquake, China
Source: PLoS One. 2011 Jun 27;6(6):e21427. doi: 10.1371/journal.pone.0021427 (PMC3124508; doi:10.1371/journal.pone.0021427)
Supplement: Table S3 — (DOC) [file pone.0021427.s003.doc]

Table S3 interactions (measured by PD value) between pairs of factors on the under-five mortality

| Interaction | intensity | collapse | slope | density | DEM | fault | geomorphology | GDP |
| --- | --- | --- | --- | --- | --- | --- | --- | --- |
| intensity |  |  |  |  |  |  |  |  |
| collapse | 0.541 |  |  |  |  |  |  |  |
| slope | 0.552 | 0.520 |  |  |  |  |  |  |
| density | 0.584 | 0.514 | 0.505 |  |  |  |  |  |
| DEM | 0.475 | 0.425 | 0.316 | 0.260 |  |  |  |  |
| fault | 0.405 | 0.370 | 0.322 | 0.156 | 0.191 |  |  |  |
| geomorphology | 0.437 | 0.342 | 0.307 | 0.189 | 0.154 | 0.155 |  |  |
| GDP | 0.524 | 0.385 | 0.301 | 0.274 | 0.230 | 0.126 | 0.151 |  |

Intensity: earthquake intensity; collapse: collapsed house; density: population density
