# Supplementary material for: Geographical Detector-Based Risk Assessment of the Under-Five Mortality in the 2008 Wenchuan Earthquake, China
Source: PLoS One. 2011 Jun 27;6(6):e21427. doi: 10.1371/journal.pone.0021427 (PMC3124508; doi:10.1371/journal.pone.0021427)
Supplement: Text S1 — Appendix. (DOC) [file pone.0021427.s004.doc]

**Appendix**

The relative risk r(*i*) for township *i* is assigned a log-normal prior distribution, log[r (i)] ~ N($\mu_{i}$,$\sigma_{i}^{2}$), where the expectation and variance are defined by a linear function of a common value (intercept), α, and two independent random effects; a heterogeneous component, e(i), that does not depend on geographic location of townships and an autocorrelated component, v(i), that reflects local spatial structure by incorporating the influence of neighboring townships. That is,

log(r(i)) = $\alpha$+ v(i) + e(i) ①

Prior distributions are then assigned to these linear terms and consequent hyperprior distributions are assigned to the variance terms as follows, thus creating a hierarchical model.

v(i) ~ N(0,κ²), e(i) ~ N(0,σ²),

v(i) | v(j); j ∈N(i) ~ N($\sum_{j=1}^{n} w*(i,j)v(j)$, κ²/$\sum_{j=1}^{n} w(i,j)$)

where $w(i,j)$ is a weights matrix element and $w*(i,j)$ is a standardized form of a weights matrix, defining the relationship between township i and its neighbor township j. The weight is defined simply as $w(i,j)$ = 1 if the two townships are adjacent (share a common border) and $w(i,j)$ = 0 otherwise.

1/κ² ~ Gamma (a, b), 1/σ² ~ Gamma (c, d)

where a and c are shape parameters, and b and d are inverse scale parameters.

Specifically, in the present study the prior distribution for the intercept $\alpha$ was assigned to a flat distribution and the hyperprior distributions for 1/κ² and 1/σ² were both specified at Gamma (0.5, 0.0005) for this study.

A single chain sampler with a burn-in of 4,000 iterations was run, followed by 1,000 iterations during which values for μ, v(i), and e(i) were stored. Diagnostic tests for convergence of the stored variables were carried out (Table 1), including the Geweke and Heidelberg-Welch tests. The tests show convergence of the chains for most of the parameters.

Table 1 Test statistics for MCMC convergence. Percentage (%) of tests passed.

| Test | $\alpha$ | $v$ | $e$ |
| --- | --- | --- | --- |
| Geweke (Z-value) | 100 | 85.34 | 84.56 |
| Heidelberg-Welch | 100 | 98.62 | 98.51 |

The Z-value threshold interval for passing is (-1.96, 1.96).
